# Expression of Low Affinity Nerve Growth Factor Receptor p75 in Classic Bladder Exstrophy

**DOI:** 10.3389/fped.2021.634343

**Published:** 2021-02-22

**Authors:** Martin Promm, Wolfgang Otto, Florian Weber, Stefanie Götz, Maximilian Burger, Karolina Müller, Peter Rubenwolf, Winfried Neuhuber, Wolfgang H. Roesch

**Affiliations:** ^1^Department of Pediatric Urology, Clinic St. Hedwig, University of Regensburg, Regensburg, Germany; ^2^Department of Urology, Caritas-St. Josef Medical Center, University of Regensburg, Regensburg, Germany; ^3^Department of Pathology, University of Regensburg, Regensburg, Germany; ^4^Center for Clinical Studies, University Medical Center Regensburg, Regensburg, Germany; ^5^Department of Urology, University Medical Center Frankfurt, Frankfurt, Germany; ^6^Institute of Anatomy and Cell Biology, Friedrich-Alexander University of Erlangen-Nürnberg, Erlangen, Germany

**Keywords:** bladder exstrophy, hyperalgesia, nerve growth factor, immunohistochemistry, pain

## Abstract

Successful primary closure of classic bladder exstrophy (BE) is crucial for development of bladder capacity and voided continence. It is universally agreed that an intensive pain management including the use of caudal epidural anesthesia is an essential cornerstone for the outcome of this complex surgery. Whether and to what extent pain is caused by structural or functional changes is not yet known. The nerve growth factor (NGF) is regarded as a marker for pain in different bladder disorders. This prospective study investigated the role of histological alterations and NGF in patients with BE including 34 patients with BE and 6 patients with congenital vesicoureterorenal reflux (VUR) who served as controls. Between January 2015 and April 2020 transmural bladder biopsies were taken from the posterior bladder wall during delayed primary bladder closure. The samples were stained for histological evaluation and subjected to immunohistochemistry to analyze NGFR p75. Differences in histological alterations were examined with Fisher's exact test, and Mann-Whitney-U-test was used to compare the NGFR p75 staining intensity between patients with BE and controls. Patients with BE showed significantly more often acute inflammation (*p* < 0.001), squamous metaplasia (*p* = 0.002), and cystitis glandularis (*p* = 0.005) as well as NGFR p75 in the urothelium (*p* = 0.003) than patients with VUR. A limitation of this study is the small number of participants due to the rare disease entity. Similar to other painful bladder disorders, pain transmission in BE after intitial closure may in part be facilitated by elevated NGF signaling through its receptor.

## Introduction

Successful primary closure is crucial for the development of bladder capacity and voided continence (1, 2). It is universally agreed that an appropriate peri- and postoperative pain management including the use of caudal epidural anesthesia is essential for the outcome of this complex surgery (3–5).

In a majority of unclosed bladders urothelial differentiation changes could be shown (6). These findings may result in a dysfunctional barrier of the urothelium with implications for the structural and functional properties of the unclosed bladder wall.

In the past decades, biomarkers have become the focus of investigations for the presence and progression of painful bladder conditions (7, 8). Several studies on urinary bladder disorders such as overactive bladder, interstitial cystitis, or neurogenic bladder have already indicated the crucial role of the nerve growth factor (NGF) in this context which has been verified by elevated NGF-levels either in the urine or in bladder wall biopsies (7–14). NGF belongs to the neurotrophin family and is considered to be involved in regulating neural function, inflammatory processes, as well as pain mechanisms (15). NGF is produced by the bladder smooth muscle and the urothelium (13). In urinary bladders, NGF is assumed to mostly affect afferent fibers. NGF also seems to play a significant role in abnormal afferent signaling and in increased bladder sensations (12, 13). NGF can activate two specific receptors: the tyrosine kinase receptor A (Trk-A) and the p75 neurotrophin receptor (NGFR p75). NGFR p75, which is present in many different types of bladder cells, is well-established in animal models and also used in clinical studies (16–18). Whether and to what extent peri- and postoperative pain might be caused by structural or functional changes in the exstrophic bladder wall is yet unknown.

Therefore, besides the histological changes, in this study we want to assess the occurrence and the distribution of low affinity receptor NGFR p75 in the bladder wall of unclosed BE.

## Materials and Methods

**Figure 1:**
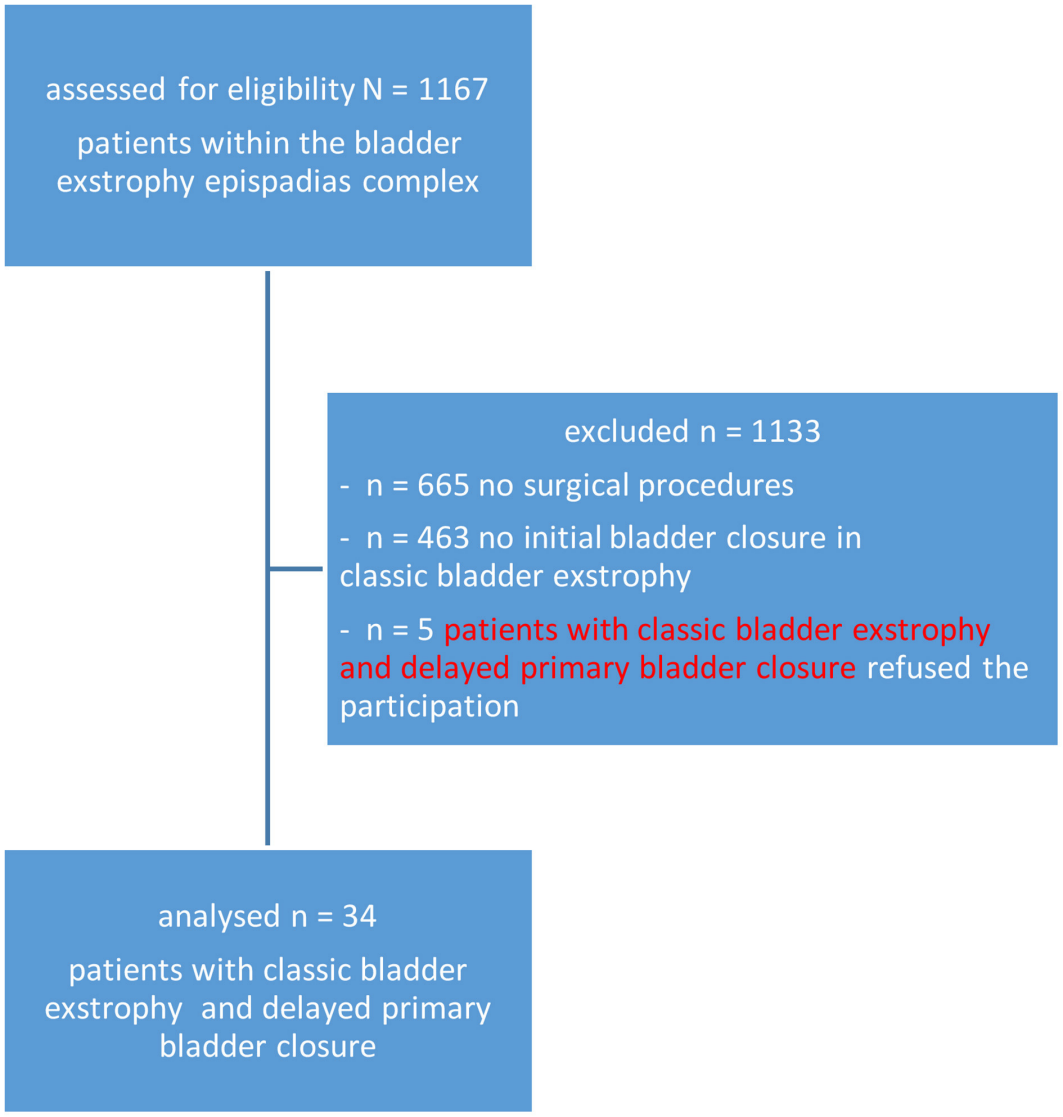
Flow-chart of patients.

Transmural bladder biopsies were prospectively obtained from patients with BE who underwent primary bladder closure in the 6–8 week of life in a single institution between January 2015 and April 2020. Only patients with classic BE were included in this investigation. Cloacal exstrophies, exstrophy variants, isolated epispadias or redo-cases were excluded (Figure 1). The biopsies from the bladder wall contained urothelium, submucosa, and detrusor muscle. Bladder biopsies from patients with congenital VUR taken during the same time period served as controls, as primary VUR is not associated with any major alterations of the urothelium and also not known for causing bladder pain (19). Only six parents had given their consent for this procedure. The biopsies were obtained during open bladder surgery.

Collection and histological as well as immunohistochemical examination were approved by the Ethics Committee of the University of Regensburg. Written informed consent was given by the parents.

### Histological Assessment

The samples were fixed in 4% formalin and sent to the laboratory. There, the tissues were embedded in paraffin and stained with hematoxylin and eosin (HE) for histological assessment by an expert uropathologist (FW). All tissues were anonymized and analyzed microscopically (Leitz DM RBE light microscope, Leica, Germany) for inflammatory infiltrates, acute and chronic inflammation, suburethelial fibrosis, squamous metaplasia, as well as cystitis glandularis and cystitis cystica.

### Immunohistochemistry

Formalin-fixed and paraffin-embedded tissue blocks were sectioned at 4 μm and mounted onto poly-L-lysine-coated slides. Immunohistochemical incubation was carried out in a BenchMark IHC Full System immunostainer (Roche Diagnostics, Mannheim, Germany) using the avidin-biotin peroxidase method with diaminobenzidine as chromogen according to the manufacturer's instructions (UltraView DAB Detection Kit, Firma Roche Diagnostics, Mannheim, Germany). Anti-NGFR p75 (rabbit polyclonal, Sigma, Germany) was used as primary antibody at a dilution of 1:400. Anonymized sections were independently evaluated semiquantitatively in a light microscope by a neuroanatomist (WN) (Leica Aristoplan, Leica, Bensheim, Germany) and a pediatric urologist (MP) (Zeiss Lab A1, Zeiss, Jena, Germany) who were both blinded to the patients' history. Density of NGFR p75 was assessed from absent, sparse, moderate dense, to dense.

### Statistical Analysis

Statistical analysis was done using the software package SPSS (Version 25, SPSS Inc, Chicago, Illinois). The level of significance was set at *p* ≤ 0.05 for all tests. Because data analyses were of exploratory manner, no adjustments for multiple testing were conducted.

Descriptive analyses were done using frequency (n), percentage (%), mean (m), standard deviation (SD), median (med), and first as well as third quartiles (IQR). Fisher-Exact tests were used to compare the two patient groups for each feature of the histopathological findings. Mann-Whitney-U-test was used to compare NGFR p75 between the two patient groups.

## Results

### Patient Data

The biopsies were prospectively obtained from 34 patients (20 boys/14 girls) with BE who underwent primary bladder closure between January 2015 and April 2020. Mean age at surgery was 61 days (SD 23.9 days, min = 38, max = 169). As controls, bladder biopsies from six children (two boys/four girls) with high-grade VUR were used. Mean age at surgery was 412 days (SD 292.4 days, min = 8, max = 822). Biopsies were taken during creating an incontinent vesicocutaneostomy and open anti-reflux plasty.

### Histological Analysis

**Figure 2:**
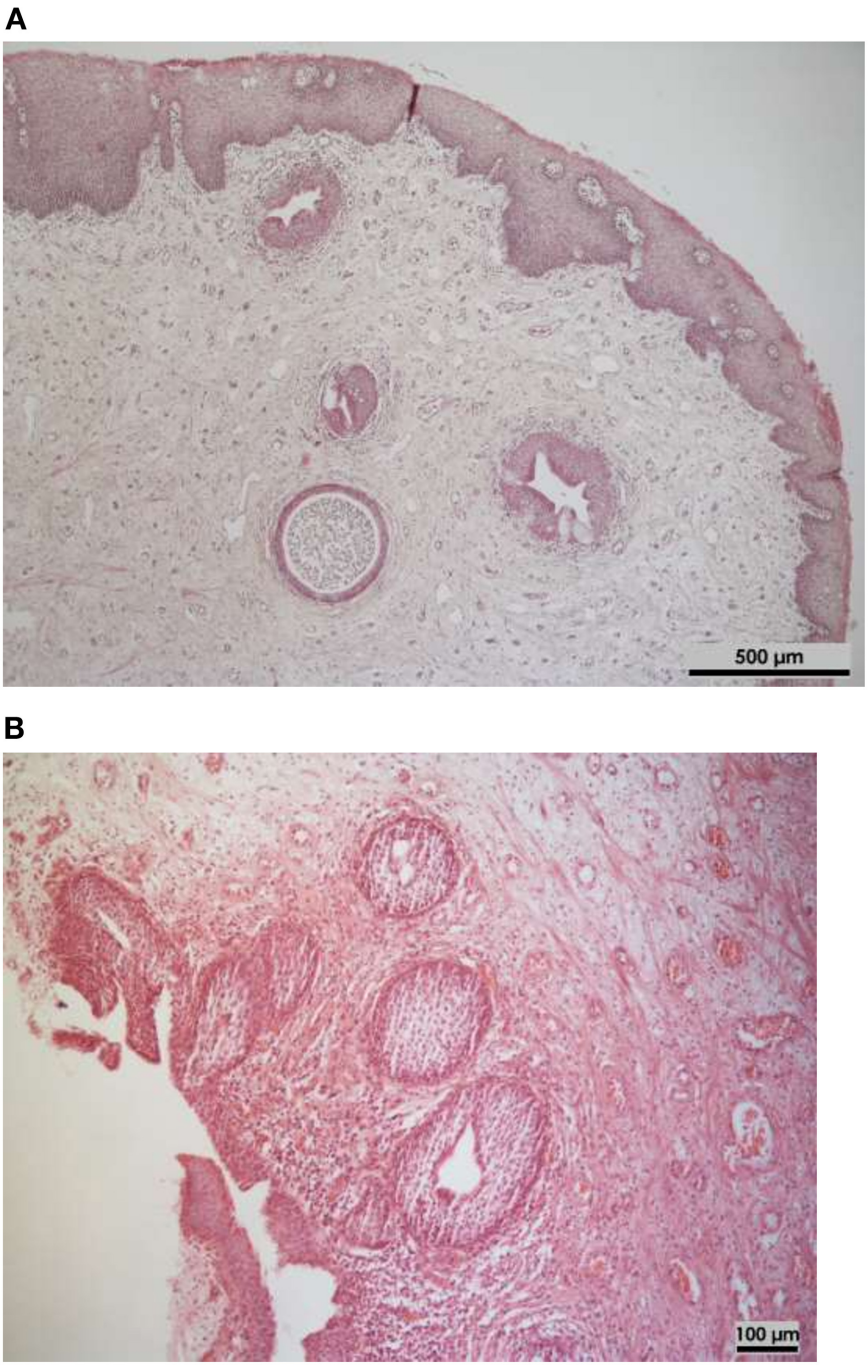
Histological assessments of bladder specimens of infants taken at primary delayed closure (Hematoxylin-eosin stained). **(A)** Typical alteration of cystitis glandularis et cystica (5× magnification). **(B)** Cystitis glandularis with squamous metaplastia and acute erosive inflammation as well as fibrosis (10× magnification).

We compared the histopathological changes in the bladder biopsies obtained from patients with BE during primary bladder closure and from patients with VUR during reflux surgery (Table 1). Acute inflammation (*p* < 0.001), squamous metaplasia (*p* = 0.002), as well as cystitis glandularis (*p* = 0.005) (Figure 2) were more frequently diagnosed in patients with BE than in patients with VUR. The two patient groups did not significantly differ in the incidence of chronic inflammation, suburethelial fibrosis, and cystitis cystica (*p*-value >0.05).

**Table 1:** Histopathological findings in bladder specimens from patients with bladder exstrophy (BE) obtained during primary closure and from patients with congenital vesicoureterorenal reflux (VUR) obtained during reflux surgery who served as controls.

	**Acute inflammation**	**Chronic inflammation**	**Subepithelial fibrosis**	**Squamous metaplasia**	**Cystitis cystica**	**Cystitis glandularis**
patients with BE	29 (85)	33 (97)	25 (74)	29 (85)	13 (38)	22 (65)
patients with VUR	0 (0)	6 (100)	5 (83)	1 (17)	0 (0)	0 (0)
*p*-value	** <0.001**	1.000	1.000	**0.002**	0.152	**0.005**

*Frequency and percentage in brackets of patients with histopathological findings are presented. Statically significant p-values of Fisher's exact tests in bold*.

### Immunohistochemical Analysis

Immunostaining for NGFR p75 in the urothelium and bladder stroma/detrusor was compared between patients with BE and patients with VUR. Table 2 summarizes the observations of NGFR p75. Patients with BE had a significantly higher density of NGFR p75 in the urothelium than patients with VUR (*p* = 0.003). The density of NGFR p75 in the bladder stroma/detrusor did not significantly differ between the two patient groups (*p* = 0.425). However, patients with BE tended to have a higher density of NGRF p75 in this area.

**Table 2:** Observation of NGFR p75 in the urothelium and detrusor in biopsies from patients with bladder exstrophy (BE) obtained during primary closure and from patients with congenital vesicoureterorenal reflux (VUR) obtained during reflux surgery who served as controls.

		**NGFR p75**				
	**Absent**	**Sparse**	**Moderate dense**	**Dense**	***p*-value**	
Urothelium	Patients with BE	1 (3)	12 (35)	12 (35)	9 (26)	**0.003**
	Patients with VUR	4 (67)	1 (17)	1 (17)	0 (0)	
Detrusor	Patients with BE	0 (0)	10 (29)	15 (44)	9 (26)	0.425
	Patients with VUR	0 (0)	2 (33)	4 (67)	0 (0)	

*Frequency and percentage in brackets are presented. Statically significant p-values of Mann-Whitney-U-tests in bold*.

**Figure 3:**
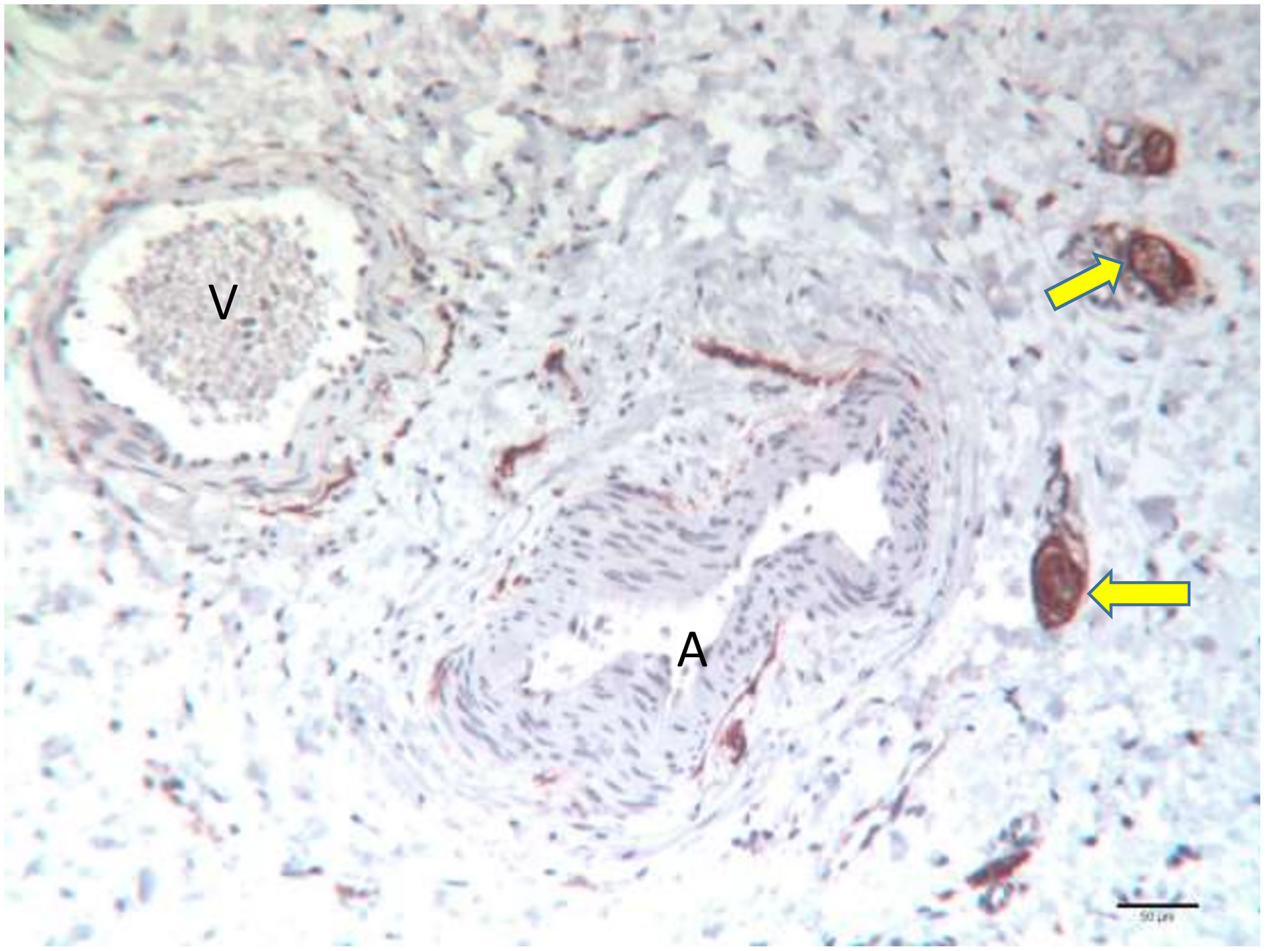
Specimen of a 54-day old girl with classic bladder exstrophy undergoing primary closure. Strong NGFR p75 positive axon bundles in the adventitia of an artery (A) and a vein (V) in the detrusor. Note the strong perineurial staining of the thicker bundles (arrow).

In the detrusor, NGFR-positive axon bundles were typically seen in the adventitia of blood vessels. Immunostaining was most pronounced in the perineurium in all patients, whereas nerve fibers sometimes showed less intense staining. At light microscopy resolution, it was impossible to determine if Schwann cells or axons or both were stained (Figure 3).

**Figure 4:**
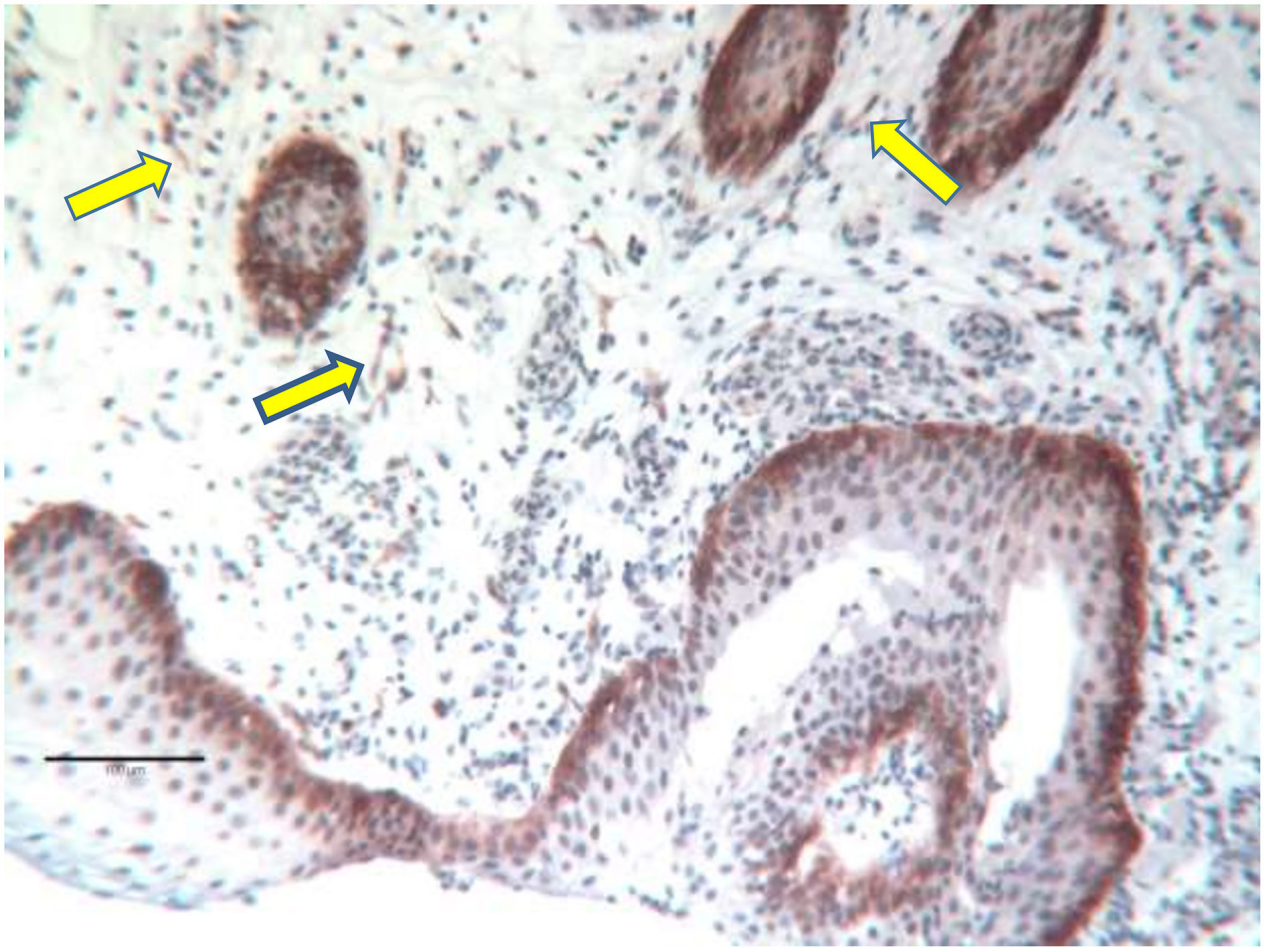
Specimen of a 64-day old girl with classic bladder exstrophy undergoing primary closure. Strong NGFR p75 positive cells in the basal urothelium. The arrows mark the thin NGFR p75 positive nerve fiber bundles of the lamina propria.

In the urothelium, NGFR p75 immunoreactivity was almost exclusively confined to the basal cell layer. Only occasionally was filamentous staining detected in the transitional layer that eventually reached the luminal layer (Figure 4).

## Discussion

Initial bladder closure in BE patients still remains a challenge. Patients undergoing this complex surgery depend on a multimodal pain management to ensure well-being and a successful outcome. Painful bladder disorders in other conditions (e.g., mucosal inflammation, interstitial cystits) are characterized by an increase in NGF and its low-affinity receptor NGFR p75 (9–12, 14). In this study we looked on the occurrence of NGFR p75 and histological alterations in 34 patients with BE presenting for delayed primary closure and six patients with VUR who served as controls. The two groups significantly differed with regard to acute inflammation (*p* < 0.001), squamous metaplasia (*p* = 0.002), and cystitis glandularis (*p* = 0.005) as well as for NGFR p75 immunostaining in the urothelium (*p* = 0.003).

Except for chronic inflammation and subepithelial fibrosis, the histological results of this study are comparable to the findings of Rubenwolf et al. who analyzed 29 patients with BE presenting for delayed primary closure (6). The two alterations may be similar in both groups due to inflammatory processes over a certain period of time. The high rate of acute inflammation and morphological changes may be related to exposure to wound dressing before bladder closure or to intrauterine amniotic fluid during exposure (6, 20). In context with bladder extrophy cystitis cystica is commonly considered as benign, whereas cystitis glandularis is discussed to have a malignant potential, but so far there is no ultimate evidence to suggest that this alteration is *per se* premalignant. (6, 21–23).

In the following sections, the role of NGF in inflammation will be discussed.

Only a few studies have immunohistochemically evaluated NGF or its low-affinity receptor p75 in bladder wall biopsies of patients with painful or irritative bladder disorders.

In 1997, Lowe et al. (12) showed increased NGF levels in 12 women with bladder conditions due to idiopathic sensory urgency, chronic cystitis, or interstitial cystitis (IC). Two bladder biopsies of the lateral bladder wall had been obtained from each patient by means of a transurethral punch. The samples were histologically graded after staining with HE. The level of NGF was assessed with fluorometric ELISA, and NGF-immunostaining was done by means of indirect immunofluorescence. The level of NGF was statistical significantly higher in the groups with bladder disorders than in the control group consisting of four women with stress incontinence without any irritative symptoms. Also, in these groups positive immunostaining for NGF was observed in the transitional epithelium, but not in the control samples. NGF-immunoreactivity was intense and present throughout all layers of the epithelium in the women with idiopathic sensor urgency, whereas the patients with chronic cystitis showed weaker staining and irregular distribution within the epithelium. In our study, NGFR p75 immunoreactivity was almost confined to the basal layer. Furthermore, in contrast to our observation, Lowe et al. did not report any positive NGF staining in the submucosal tissue or in muscle layers (12).

In 1998, Vaidyanathan et al. published their results about the occurrence of the p75 NGF receptor in the urothelium in 26 adult patients with neuropathic bladders. Two cold cup biopsies were cystoscopically obtained from the trigonal area of each patient. One biopsy was referred to routine histopathological examination, and the other one was stained immunohistologically for NGFR p75. In all patients, immunostaining for NGFR p75 was detected in neural structures and, similar to our results, especially in the basal layer of the transitional layer. In 12 patients, immunostaining of the luminal layer showed varying degrees. In many stains, the nerve fibers in the submucosa ran in a random manner, also reaching very close to the basal layer, but no intra-epithelial terminals were seen (14).

In 2013, scientists from York examined tissue samples obtained cystoscopically either as cold cut biopsies or from cystectomy specimens of 21 patients with the painful bladder condition of ketamine cystitis (KC). The tissues were immunohistochemically labeled for NGFR p75. The findings of the 21 patients with KC were compared to those of six patients with idiopathic detrusor overactivity (IDO), four patients with stress urinary incontinence (SUI), and 11 patients with IC.

NGFR p75 was predominantly detected basally in the samples of patients with KC, but irregularly also in the samples of patients with IDO and SUI. Eight of 11 samples of patients with IC did not show the normal basal pattern but weak punctuate staining throughout the urothelium. In 16 patients with KC, urothelium and intermediate cells were retained, and 10 of the 16 samples showed an expansion of intense basal NGFR p75 expression into the intermediate cells (24).

There are still many unresolved issues concerning the role of NGF and its receptor NGFR p75 in the pathophysiology of bladder disorders. Our results showed a statistically significant difference in NGFR p75 in the urothelium between patients with BE and patients with VUR. No statistically significant difference was found in the submucosal tissue and detrusor but a notable tendency to higher density.

Increased distribution of NGF or NGFR p 75 in the urothelium is more often reported in painful bladder disorders, whereas higher levels of NGF or NGFR p75 have been described for the submucosal tissue and detrusor in functional bladder alterations (12–14, 24).

In all our specimens, NGFR p75 in the submucosal layers including the detrusor was most pronounced in the perineurium, whereas nerve fibers showed less intense staining. This finding is consistent with the observation in human cutaneous nerve fibers in which NGFR p75 mainly occurs in Schwann cells, particularly in their membrane (25). Abdo et al. even found specialized cutaneous Schwann cells that are responsible for initiating pain (26). The distribution of NGFR p75 on a cellular level based on electron microscope findings may be helpful to clarify its role in the mechanism of pain regulation.

Based on numerous experimental and clinical studies, there is no longer any doubt about the correlation between NGF and painful sensations or inflammatory processes (13, 27, 28). As mentioned above, our histological findings show a significant difference in acute inflammation and cystitis glandularis between patients with BE and the control group. Inflammation has been discussed to stimulate NGF production (13). NGF acts as a mediator inducing clinical symptoms such as thermal and mechanical hyperalgesia; even a single systemic injection of NGF induces profound and long-lasting hyperalgesia (27, 28). Lewin et al. (28) could even demonstrate that NGF-induced thermal and mechanical hyperalgesia are mediated by different mechanisms. The rapid onset of thermal hyperalgesia is due to peripheral mechanisms with mast cell degranulation, and the late component involves central N-methyl-D-aspartate receptors (NMDA), whereas NGF-induced mechanical hyperalgesia seems to be independent of both (28).

NGF seems to be key factor in painful and functional bladder disorders. Although no data exist on NGF or its p75 receptor in BE, our results are in line with investigations showing increased NGF expression of the unclosed bladder wall in patients with other painful urological disorders (7–13, 24).

Further studies also in closed bladders and including electron microscopy are necessary to detect the distribution of NGFR p75 in the bladder wall. Additionally, clinical trials are of utmost importance to evaluate the relevance of NGFR p75 and NGF as potential biomarkers in this rare disease entity. In this context, monitoring of potential therapeutic approaches such as antimuscarinics or hyaluronic acid is of particular interest, even in unclosed bladder templates (29).

This study has some limitations. First, because of the staining for NGFR p75 used in this study, the results have to be interpreted carefully when drawing any conclusions on NGF itself. However, other studies have indicated a correlation between the expression of NGF and its low-affinity receptor p75 (18, 30).

Second, this study did not assess the level of pain or the need for analgesics. Therefore, it remains unclear whether it is the increased level of NGF or its receptor p75 that actually leads to a higher level of pain in the respective patient group.

Third, our study only included patients with unclosed BE undergoing delayed bladder closure. Further studies should include samples from neonates undergoing immediate closure as well as from closed bladders to determine possibly varying presence and distribution patterns of the receptor in these groups.

Lastly, the small sample size of this study precludes advanced statistical comparison but represents a comparatively large sample for this rare disease entity.

Although there is a number of limitations, we believe that this study might contribute to possibly identify a potential pain pathway in this rare entity.

## Conclusion

This study is the first to assess histological alterations as well as the occurrence and distribution of NGFR p75 in the unclosed exstrophic bladder wall. We hypothesize that structural or functional changes in the exstrophic bladder wall may contribute to peri- and postoperative pain. This hypothesis can be supported by a statistically significantly increased occurrence of acute inflammation as well as a statistically significantly increased occurrence of NGFR p75 in the urothelium in patients with unclosed bladder templates compared to patients with VUR.Nevertheless, our data are not sufficient proof to clarify the role of these changes regarding this topic. Further studies, especially in neonatal closures as well as in long term of closed bladders are crucial to substantiate these first findings and to evaluate the possible relevance of NGF as potential biomarkers or potential therapeutic approaches in this rare entity.

## Data Availability Statement

The original contributions presented in the study are included in the article/supplementary material, further inquiries can be directed to the corresponding author.

## Ethics Statement

The studies involving human participants were reviewed and approved by Ethikkommittee der Universität Regensburg. Written informed consent to participate in this study was provided by the participants' legal guardian/next of kin.

## Author Contributions

MP contributed to the data analysis, evaluation of the immunohistochemical-stained section, evaluation of the results, manuscript writing, and the revision and submission of the manuscript. SG contributed to manuscript writing and embedding and staining of the samples. WO contributed to the data analysis, conducted the statistics, evaluation of the results, and writing and revision of the manuscript. FW contributed to the data analysis, evaluation of the results, histological assessment, and manuscript writing. KM contributed to the statistical data analysis. WN contributed to the data analysis, evaluation of the immunhistochemically stained sections, and took the photographs, evaluation of the results and writing of the manuscript, and interpreted the study findings. WR designed the study, contributed to the data analysis, conducted surgery, obtained the biopsies, and interpreted the study findings. MP, WO, FW, MB, PR, KM, WN, and WR critically evaluated the manuscript and approved the final manuscript. All authors contributed to the article and approved the submitted version.

## Conflict of Interest

The authors declare that the research was conducted in the absence of any commercial or financial relationships that could be construed as a potential conflict of interest.

## Abbreviations

BE: classic bladder exstrophy
BOO: bladder outlet obstruction
HE: hematoxylin and eosin
KC: ketamine cystitis
IC: interstitial cystitis
NB: neurogenic bladder dysfunction
NDMA: N-methyl-D-aspartate receptors
NGF: nerve growth factor
NGFR p75: low affinity nerve growth factor receptor p75
OAB: overactive bladder
STD: standard deviation
Trk-A: tyrosine kinase receptor A
VUR: vesicoureterorenal reflux.
